# The distribution and epidemic characteristics of cerebrovascular disease in followed-up hypertension patients

**DOI:** 10.1038/s41598-021-88127-5

**Published:** 2021-04-30

**Authors:** An-le Li, Shuai Zhu, Zhi-hao Hu, Qian Peng, Xiang Fang, Yi-ying Zhang

**Affiliations:** Jiading District Center for Disease Control and Prevention, Shanghai, China

**Keywords:** Diseases, Medical research

## Abstract

To explore distribution and epidemic characteristics of CVD in followed-up HP patients. Using the Hypertension Follow-up Management System database in Jiading district in Shanghai. We designed a retrospective cohort study that included all followed-up hypertension patients between 2002 and 2020. The endpoint was the occurrence of CVD confirmed by the hospital; otherwise, the patients were tracked until September 30, 2020. Record information of every patient has been collected in the registration card and each followed-up record. Among 223,097 observational followed-up HP patients, the total number of person years of observation was 4,244,421.25 person-year, 11,143 patients had developed CVD from hypertension before the deadline, the total incidence density was 0.00263 per person-year (male 0.00292; female 0.00238) and the complication ratio of CVD in HP patients was 4.99% (male 5.25%; female 4.76%) during follow-up period. The proportion of ischemic cerebrovascular, hemorrhagic cerebrovascular and unclassified stroke was respectively 71.18%, 5.95% and 22.87% in hypertensive CVD. Complication ratio of CVD increased with age, the group under 30 was 0, and the group over 70 was the highest (6.90%). The complication ratio of grad I, grad II and grad III blood pressure were respectively 4.79%, 4.96% and 6.13%. The complication ratio was 4.92% in only high systolic blood pressure patients; 17.23% in only high diastolic blood pressure patients; 4.59% in high systolic and diastolic blood pressure patients. The peak of complication ratio of CVD was 9–10 years after the registered and followed-up. The proportion of CVD cases in HP patients from April to June was the largest in the four seasons; the proportion of patients from October to December was the minimum. HP patient was prone to falling cerebrovascular disease; the main type of disease was cerebral infarction. Complication ratio in male incidence was higher than that in female. The complication ratio of CVD increased with age, blood pressure and duration of HP patients. It had seasonal characteristics, which was relatively high from April to June within year.

## Introduction

A recent study showed that the standardized prevalence of stroke among residents aged 40 or above increased from 1.89% (2012) to 2.32% (2018), the incidence rate of residents among 40–74 years old was increasing with an average annual rate of 8.3% in China^1,2^. The latest reports of GBD (Global Burden of Disease Study) showed China's overall lifetime risk of stroke was 39.9%, ranking the first in the world, which means that about two out of five people would suffer from stroke in their lifetime^3,4^. It’s reported that hypertension led 10.4 million deaths worldwide, it was effectively associated with stroke and worse prognosis, and it was the first modifiable vascular risk factor^5^. Most of the incidence of SVD related strokes might cause by raised blood pressure^6^. The burden of cerebrovascular disease increased with the increase of the number of the elderly. Hypertension, diabetes, dyslipidemia, diet, smoking and physical activity played efficient role in stroke related diseases^2,5,6^.

In order to better observe and explore the regular characteristics between hypertension and cerebrovascular disease, a large long term follow-up cohort of hypertension patients have established in Shanghai China. It was designed to mainly explore regular pattern of the main population of hypertensive patients with cerebrovascular disease, the time of occurrence, the type of lesions and so on. The purpose of this study was to explore the baseline characteristics of cerebrovascular diseases in hypertension, and the association between hypertension and cerebrovascular diseases base on a large long term follow-up cohort hypertension patients.

## Methods

### Data sources

**Figure 1:**
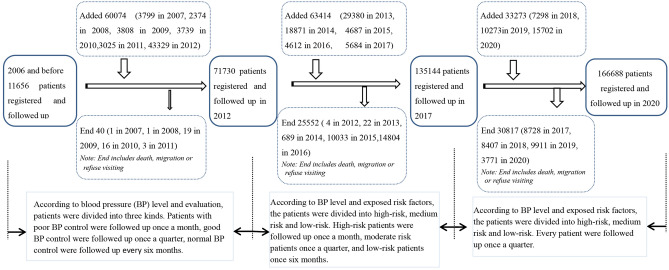
Dynamic changes of follow-up management cohort of hypertension in Jiading Shanghai.

The Hypertension Follow-up Management System was officially built and implemented in Jiading district in Shanghai China in 2002. The patients with hypertension in community were registered, investigated, filed and followed up regularly by community health workers or family doctors in Jiading district in Shanghai. The Hypertension Follow-up Management System Database records the medical information of all registered and followed-up hypertension patients. 223,097 patients were registered and followed-up in totally, and 56,409 HP patients dropped out of the cohort due to death or emigration or so on during this period, 166,688 patients were entered into the end time of the observation. All patients registered and followed-up entered the observation queue, and the cohort of hypertension patients was dynamic. Dynamic changes of the HP cohort see Fig. 1. All about cerebrovascular disease occurred in patients must be recorded in detail. Cerebrovascular disease must be diagnosed and confirmed by a senior hospital. The study was approved by Science and Technology Commission, Health Commission and CDC in Jiading district in Shanghai. All methods were carried out in accordance with relevant guidelines and regulations.

### Study objects and data collection

All study objects were from the above Hypertension Follow-up Management System, and observation deadline of data of study objects were collected was September 30, 2020. Clinical information and biological information for each hypertension patient in the registration card and each followed-up record were collected. Their socio- demographic status (birth date, gender, habitation, occupation and education level), etiology of family history, disease history (disease kind, diagnosed date and diagnosed hospital), lifestyle (smoking, drinking and exercise) and blood pressure and so on were documented. Objects of hypertension patients in this study were primary hypertension, excluding secondary hypertension patients. Cerebrovascular disease refers to that the onset date was later than the date of hypertension diagnosed and registration, Cases were excluded if onset date was before or close (within one month) the date of hypertension diagnosed and registration.

### Definition and classification

According to the classification of diseases and ICD-10 coding rules, and to facilitate this statistical analysis, cerebrovascular diseases mainly included ischemic cerebrovascular diseases, hemorrhagic cerebrovascular diseases and unclassified stroke (I64) in this study. Ischemic cerebrovascular diseases include transient ischemic attack (G65), cerebral infarction (I63). Hemorrhagic cerebrovascular diseases included subarachnoid hemorrhage (I60), intracerebral hemorrhage (I61) and other non-traumatic intracranial hemorrhage (I62).

Observation starting point: refers to the time point when hypertension patients were registered and entered the follow-up management queue. Observation end point: refers to the time when hypertension patients have expected outcome events (stroke) or withdraw from the observation queue due to loss of follow-up. Follow up time or observation duration: refers to the time difference between the end point and the starting point (observation end point minus observation starting point). Censored values were calculated for all study populations that did not reach the observed end point.

### Statistical analysis

All databases in the Hypertension Follow-up Management System were exported into Microsoft Excel database according to the cut-off time prescribed above, and then the relevant logical examination, data screening and conversion were carried out. Statistical analyses were performed using the statistical software package (IBM SPSS statistics version 21). Mean and standard deviation (SD) were used to compute for quantitative variables, and comparisons between groups were performed by t-test. Number (n) and percentage (%) were computed for the categorical data, comparisons between groups were performed by the chi-square (χ^2^) test. All results were statistically significant if the two tailed p value was less than 0.05. The life table method (step size was 1 year) used to calculate the person years, for further calculate incidence density.

### Ethics approval and consent to participate

Ethical approval was granted by Jiading district center for disease control and prevention research ethics committee. All subjects gave informed consent to participate in the study, they would like to participate in registry and manage and answer all the related questions in the follow-up.

## Results

### Baseline demographic and clinical characteristics

223,097 hypertension patients (107,409 male, 115,688 female; mean age: 62.39 ± 12.18 years old) were registered and followed up, 166,688 hypertension patients (80,251 male, 86,437 female; mean age: 63.16 ± 11.22 years old) were entered into the end time of the observation. By counting the censored values, the total number of person years of observation was 4,244,421.25 person year (male 1,930,489, female 2,313,932.25). Among these observational HP patients, the blood pressure value at the time of registry: systolic blood pressure was 154.49 ± 13.83 mmHg; diastolic blood pressure was 92.57 ± 8.74 mmHg. Overall, 11,143 HP patients had developed CVD from hypertension before the deadline, the total incidence density was 0.00263 per person-year (male 0.00292; female 0.00238) and the complication ratio of CVD in HP patients was 4.99% (male 5.25%; female 4.76%). The mean age of HP patients complicated by CVD was 73.44 ± 10.39 years old and the average observation duration or followed up time was 5.83 ± 3.95 years.

**Figure 2:**
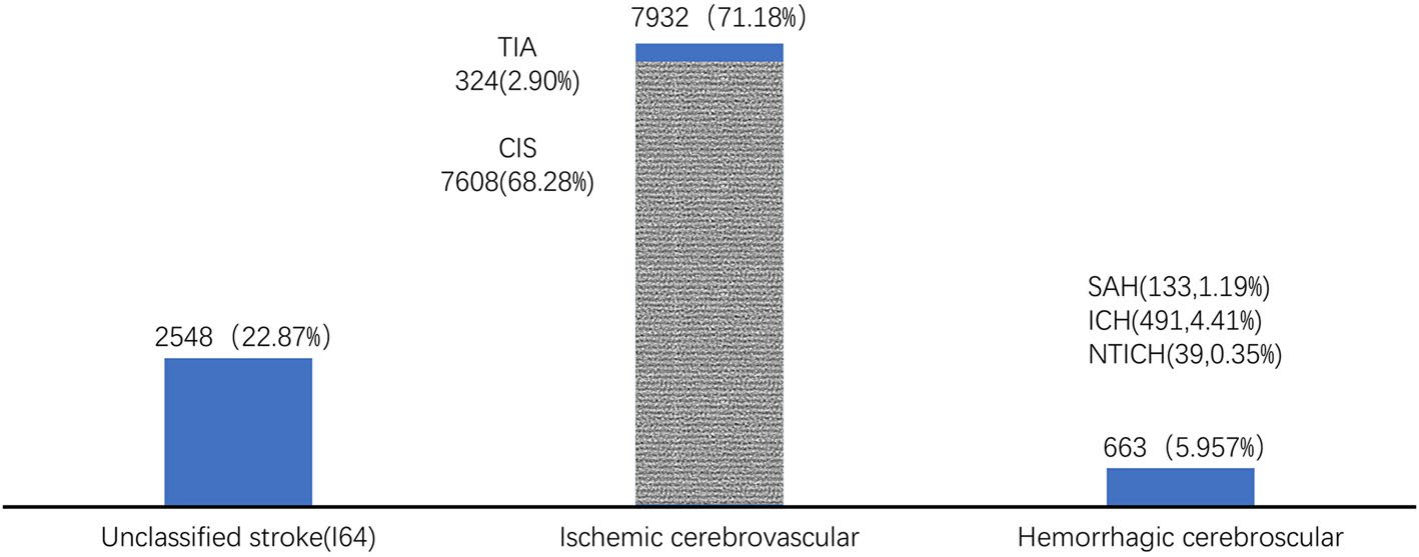
Types, numbers and ratio of CVD in hypertension patients.

Figure 2 showed that the types and numbers of 11,143 CVD in HP patients, 7932 cases were ischemic cerebrovascular (the constituent ratio was 71.18%); 663 cases were hemorrhagic cerebrovascular (the constituent ratio was 5.95%); 2548 cases were unclassified stroke (the constituent ratio was 22.87%). In the ischemic cerebrovascular, there were 7609 (the constituent ratio was 68.28%) cerebral infarction (CIS, I63), 323 (the constituent ratio was 2.90%) transient ischemic attack (TIA, G65). In the hemorrhagic cerebrovascular, there were 133 (the constituent ratio was 1.19%) subarachnoid hemorrhage (SAH, I60), 491 (the constituent ratio was 4.41%) intracerebral hemorrhage (ICH, I61), 39 (the constituent ratio was 0.02%) non-traumatic intracranial hemorrhage (NTICH, I62). The results show that ischemic cerebrovascular was the main type of stroke in patients with hypertension, prevention of cerebral infarction in patients with hypertension is the key to prevent complications.

### HP characteristics and CVD

In this followed up observational cohort of hypertension patients, the incidence density of cerebrovascular disease (CVD) in male was 0.00292 per person-year (complication ratio 5.25%, 5636/107,409), and female was 0.00238 per person-year (complication ratio 4.76%, 5507/115,688). Male was higher than female (x^2^ = 27.840, p < 0.001). Age distribution: the cases number and complication ratio of CVD in HP patients under 30 years old were 0, 30–39 years old 21 (1.50%), 40–49 years old 135 (1.79%), 50–59 years old 911 (3.14%), 60–69 years old 2492 (3.31%), over 70 years old 7584 (6.90%). There was no CVD case in the hypertension patients group under 30 years old and cumulative incidence of CVD was the highest in the age group over 70 years old. There were significant differences in the number and ratio of CVD among different age groups (x^2^ = 170.434, p < 0.001). The older HP patients were, the more likely CVD were to have. See Table 1.

**Table 1:** Cumulative incidence of cerebrovascular diseases in hypertensive patients with different sex, age and blood pressure type (n, %).

	N	Unclassified stroke (I64) n (%)	Ischemic cerebrovascular			Hemorrhagic cerebrovascular				Total n (%)	x^2^ p
			CIS (I63)	TIA (G45)	Total n (%)	SAH (I60)	ICH (I61)	NTICH (I62)	Total n (%)		
**Sex**											
male	107,409	1373 (1.28%)	3754	158	3912 (3.64%)	66	268	17	351 (0.44%)	5636 (5.25%)	x^2^ = 27.840
Female	115,688	1175 (1.02%)	3855	165	4020 (3.47%)	67	223	22	312 (0.36%)	5507 (4.76%)	p < 0.001
**Age (y)**											
< 30	54	0 (0.00%)	0	0	0 (0.00%)	0	0	0	0 (0.00%)	0 (0.00%)	x^2^ = 170.434 p < 0.001
30 ~	1401	2 (0.14%)	15	1	16 (1.14%)	0	3	0	3 (0.29%)	21 (1.50%)	
40 ~	7550	13 (0.17%)	110	3	113 (1.50%)	1	8	0	9 (0.16%)	135 (1.79%)	
50 ~	29,005	66 (0.23%)	755	40	795 (2.74%)	6	42	2	50 (0.23%)	911 (3.14%)	
60 ~	75,200	141 (0.19%)	2104	91	2195 (2.92%)	28	114	14	156 (0.28%)	2492 (3.31%)	
≥ 70	109,887	2326 (2.12%)	4625	188	4813 (4.38%)	98	324	23	445 (0.54%)	7584 (6.90%)	
**BP**											
High SBP	42,491	428 (1.01%)	1474	51	1525 (3.59%)	31	100	7	138 (0.43%)	2091 (4.92%)	x^2^ = 198.685 p < 0.001
High DBP	6094	64 (1.05%)	859	44	903 (14.82%)	14	66	3	83 (1.82%)	1050 (17.23%)	
HBP	174,513	2057 (1.18%)	5275	228	5503 (3.15%)	88	325	29	442 (0.34%)	8002 (4.59%)	
Total	223,097	2548 (1.14%)	7609 (3.42%)	323 (0.14%)	7932 (3.56%)	133 (0.06%)	491 (0.22%)	39 (0.02%)	663 (0.30%)	11,143 (4.99%)	

*TIA* transient ischemic attack, *CIS* cerebral ischemic stroke/cerebral infarction, *TIA* transient ischemic attack, *SAH* subarachnoid hemorrhage, *ICH* intracerebral hemorrhage, *NTICH* non-traumatic intracranial hemorrhage, *n (%)* number (complication rate %), *High SBP* only high systolic blood pressure, *High DBP* only high diastolic blood pressure, *HBP* high systolic and diastolic blood pressure.

Among these cerebrovascular disease in hypertension patients, the proportion of ischemic cerebrovascular, hemorrhagic cerebrovascular and unclassified stroke were respectively 71.17%, 5.95% and 22.88% of the total number of CVD. Cerebral infarction (CIS) and transient ischemic attack (TIA) were respectively 68.28% and 2.90% of the total number of CVD in ischemic CVD; subarachnoid hemorrhage (SAH), intracerebral hemorrhage (ICH) and non-traumatic intracranial hemorrhage (NTICH) were respectively 1.19%, 4.41% and 0.35% of the total number of CVD. The observation results of this hypertension cohort showed that ischemic CVD was the most important type of CVD in HP patients.

**Figure 3:**
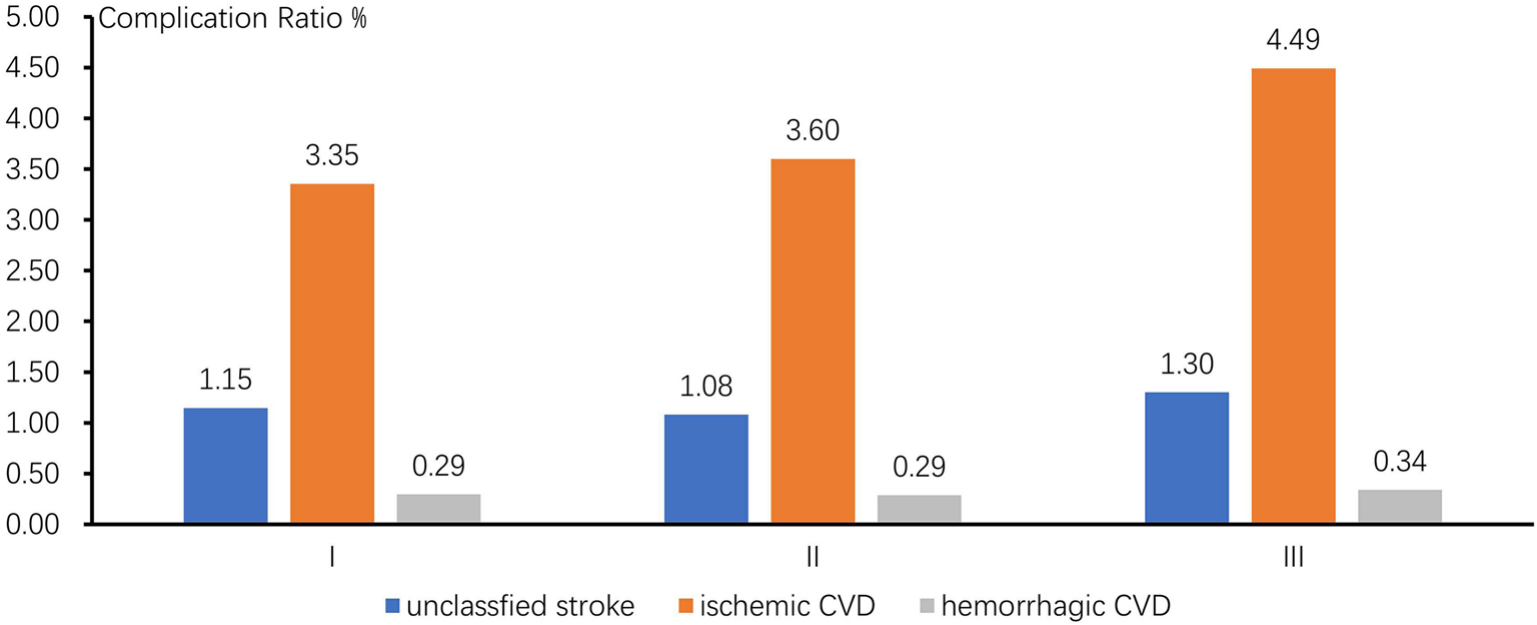
Complication ratio of CVD in different blood pressure grad.

Figure 3 showed the complication ratio of CVD was different in different grad of registered blood pressure in HP patients (x^2^ = 113.895, p < 0.001). When 140 mmHg ≦ SBP < 160 mmHg or 90 mmHg ≦ DBP < 100 mmHg (Called grad I), the complication ratio of CVD was 4.79%; 160 mmHg ≦ SBP < 180 mmHg or 100 mmHg ≦ SBP < 110 mmHg (Called grad II), the complication ratio of CVD was 4.96%; 180 mmHg ≦ SBP or 110 mmHg ≦ DBP (Called grad III), the complication ratio of CVD was 6.13%. The higher the registered blood pressure was, the higher the complication ratio of CVD was.

The complication ratio of CVD was different in different types of hypertension. See Table 1. The complication ratio of CVD was 4.92% in high SBP (only high systolic blood pressure) patients, 17.23% in high DBP (only high diastolic blood pressure), and 4.59% in HBP (systolic and diastolic blood pressure were high). The results showed that there were significant differences in the complication ratio of CVD among different types of hypertension (x^2^ = 198.685, p < 0.001). The complication ratio of CVD in high DBP was significantly higher than that in other patients.

### HP duration and CVD

Among 11,143 CVD cases in patients with hypertension, 30.38% of CVD cases occurred within 5 years of registration and follow-up, 46.79% occurred more than 5 years and less than 10 years of registration and follow-up, 20.69% occurred more than 10 years and less than 15 years of registration and follow-up, and 2.14% occurred more than 15 years and later of registration and follow-up.

**Figure 4:**
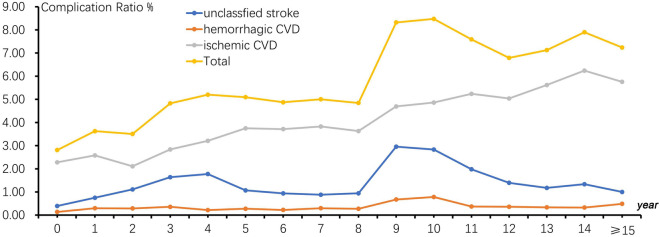
The complication ratio of CVD and the duration of HP patients.

The complication ratio of CVD in hypertension patients was different in different registration and follow-up periods. The results showed that there were significant differences in the complication ratio of CVD among different registration and follow-up periods (x^2^ = 173.546, p < 0.001).With the extension of the duration of hypertension, the complication ratio of CVD will continue to increasing. See Fig. 4. Generally speaking, the peak of complication ratio of CVD in hypertension patients was 9–10 years after the registered and followed-up. After that, complication ratio will drop slightly, and the clam will remain at a high level. The complication ratio of ischemic CVD would continue to increasing with the extension of the duration of hypertension. The complication ratio of hemorrhage CVD had been maintained at a low level, and the fluctuation range was not very large. In fact, unclassified stroke also included cerebral infarction and hemorrhage cerebrovascular disease; therefore, the changed trend of its incidence ratio was basically consistent with that of the overall cerebrovascular disease.

### Seasons distribution of CVD

**Figure 5:**
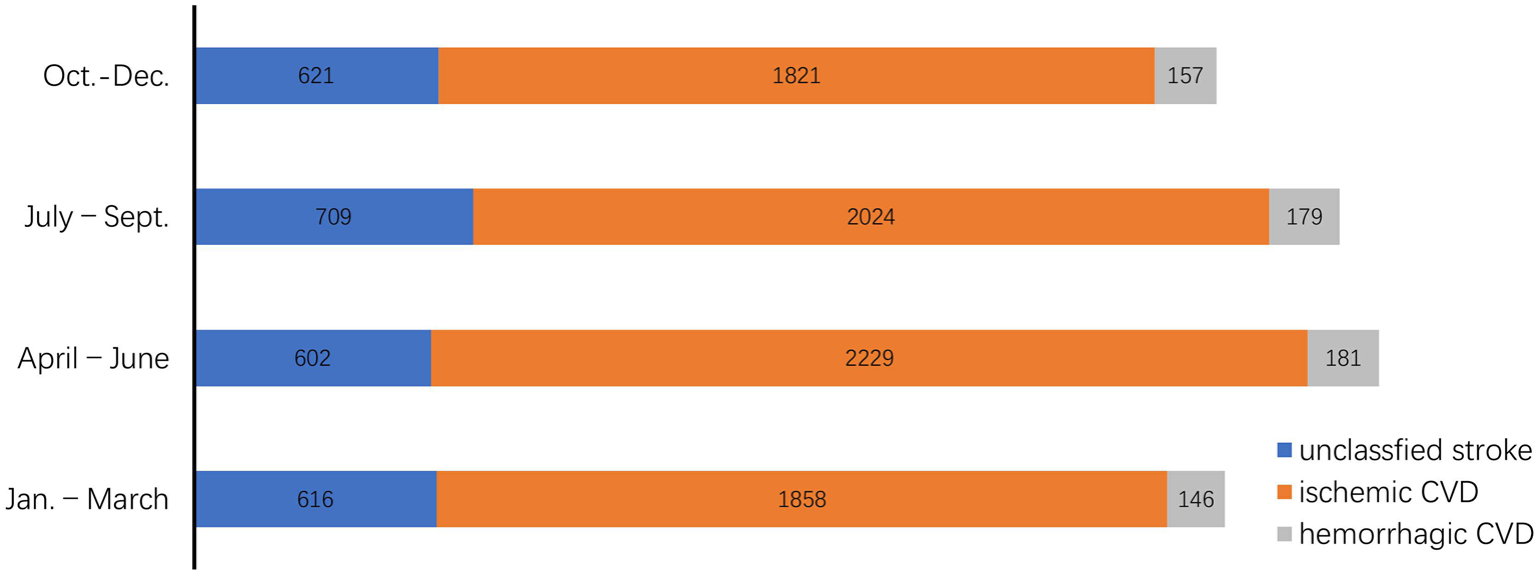
Distribution of CVD in HP patients in different seasons.

Among 11,143 CVD cases in HP patients, season distribution was different. From January to December, the proportion of CVD in HP patients were respectively 7.53%, 7.29%, 8.55%, 8.96%, 9.71%, 9.10%, 9.63%, 8.13%, 8.01%, 8.15%, 7.19% and 7.76%. The difference of the proportion of different types of CVD in different months was significant (x^2^ = 149.439, p < 0.001). The proportion of patients from January to March accounted for 23.51% of the total number of patients; that from April to June accounted for 27.03%; from July to September accounted for 26.13%; and from October to December accounted for 23.32%. The difference of the proportion of different types of CVD in different seasons was significant (x^2^ = 60.656, p < 0.001). See Fig. 5. Comparatively speaking, the number of patients of CVD from April to June was the largest in the four seasons; the next was that from July to September; the number of patients from October to December was the minimum.

## Discussion

In this study, Among 223,097 registered and followed-up hypertension patients, the total number of person years of observation was 4,244,421.25 person year, the total incidence density was 0.00263 per person-year (male 0.00292; female 0.00238). And the complication ratio of CVD in hypertension patients was 4.99% (male 5.25%; female 4.76%). The proportion of ischemic cerebrovascular, hemorrhagic cerebrovascular and unclassified stroke was respectively 71.18%, 5.95% and 22.87% in hypertensive CVD. Ischemic CVD was the most important type of cerebrovascular disease in hypertension patients in Jiading Shanghai China. What need to be explained here was that Jiading district government began to implement the policy of free medication for hypertension patients with medical insurance in rural areas as early as around 2000. Later, due to the cancellation of rural household registration and the implementation of urban population policy in Shanghai, the policy of free medication gradually faded out but still open to poor residents. Therefore, in this observation of patients with hypertension, the vast majority of patients have been taking antihypertensive drugs, only a few patients were not willing to accept drug treatment (But received some non-drug treatment, such as lifestyle adjustment and so on). Therefore, the incidence rate of stroke in hypertension patients reflected mainly the incidence of stroke in hypertension patients having been taken medical treatment. If HP patients did not take medical treatment, the frequency of stroke might be higher and the time of occurrence might be earlier.

In addition, some patients withdraw from the observation in this cohort, this part of the hypertension might also had CVD, but no added to the CVD cases, which may reduce the complication ratio of stroke in this study. For all that, the complication ratio of CVD was significantly higher than that of non-hypertensive patients people reported in the past in China^1,2,7^. The result of this long-term followed-up for hypertension showed that incidence of CVD in hypertension patients was significantly higher than that in normal population. Stroke was the first cause of death in China and the second cause of death in the world^8^. Primary prevention was particularly important because about 77% of strokes were first events^9^. Specifically, hypertension was a well-recognized major risk factor for stroke^10,11^. The kind of effective strategy for preventing stroke was controlling the risk factors of stroke^12^. Risk of stroke increased in patients with hypertensive pregnancy disorders, compared with those without disorder; the patients who had experienced the disorders had a 2.134 fold higher risk of developing stroke in the future^13^. Hypertension can cause cerebral infarction, which was the most important factor of cerebral infarction. Because long-term high blood pressure can lead to cerebral vasospasm, resulting in blood stasis, the formation of embolism^14^.

The results of long-term followed-up of HP patients showed that complication ratio of CVD increased with age, blood pressure and duration of HP patients. The complication ratio of CVD in patients under 30 years old were 0%, 30–39 years old 1.50%, 40–49 years old 1.79%, 50–59 years old 3.14%, 60–69 years old 3.31%, over 70 years old 6.90%. There was no CVD case in the hypertension patients group under 30 years old and was the highest in the age group over 70 years old. The older the age was, the higher the complication ratio was. The complication ratio of CVD of grad I hypertension, grad II hypertension and grad III hypertension, were respectively 4.79%, 4.96% and 6.13%. The complication ratio of CVD was different in different types of HP patients; complication ratio was 4.92% in only high systolic blood pressure patients; 17.23% in only high diastolic blood pressure patients; 4.59% in high systolic and diastolic blood pressure patients. With the extension of the duration of hypertension, the complication ratio of CVD will continue to increasing, the peak of complication ratio of CVD in hypertension patients was 9–10 years after the registered and followed-up. The results remind us that the risk of high diastolic blood pressure was higher than that of systolic blood pressure. Some studies showed that hypertension was the most important risk factor for stroke^10,15–17^; Hypertensive patients with > 80 bpm had the highest risk of stroke^18^. In addition, this study result showed that hypertension duration was associated with the increased prevalence of CVD, the prevalence of ischemic CVD would continue to increasing with the extension of the duration of hypertension. These research results reminded us that CVD was related to the control of blood pressure level, age, diet and drugs of patients with hypertension^19–21^.

The result in this study showed that season distribution of CVD in HP patients was different, CVD incidence varied from month to month. In terms of months, the complication ratio was relatively high from May to July. In terms of seasons, the number of patients from January to March accounted for 23.51% of the total number of patients; that from April to June accounted for 27.03%; from July to September accounted for 26.13%; and from October to December accounted for 23.32%. Comparatively speaking, the number of patients of CVD from April to June was the largest in the four seasons; the next was that from July to September; the number of patients from October to December was the minimum. It reminds us that we should pay attention to the seasonal changes in the prevention and control of hypertension in the community.

A recent study revealed that although patients were more likely to survive after stroke, poorer recovery was noted^22^. The disease of CVD, not only seriously endangers Chinese residents, but also has a serious impact on other countries or region area in the world^23–25^. Some studies told us many patients are exposed to a lot of risk factors (especially sub-health status plus bad lifestyle and living habits), but these patients did not know they were facing the risk of stroke^26–29^. Therefore, we should pay attention to the prevention and control of CVD, the prevention and control of cerebrovascular disease in patients with hypertension, especially to strengthen the prevention and control of cerebral infarction in patients with hypertension.

## Data Availability

The data that support the findings of this study are available from the Hypertension Follow-up Management System database in Jiading district in Shanghai, but restrictions apply regarding the availability of these data, which were used under license for the current study and thus are not publicly available. The data are, however, available from the authors upon reasonable request and with permission of the Jiading district health committee in Shanghai.

## Abbreviations

HP: Hypertension
BP: Blood pressure
SBP: Systolic blood pressure
DBP: Diastolic blood pressure
ICD: International classification of diseases
CVD: Cerebrovascular diseases
ICVD: Ischemic cerebral vascular disease
SAH: Subarachnoid hemorrhage
ICH: Intracerebral hemorrhage
CIS: Cerebral ischemic stroke/cerebral infarction
TIA: Transient ischemic attack
NTICH: Non-traumatic intracranial hemorrhage
CI: Cumulative incidence
